# The effects of observation of walking in a living room environment, on physical, cognitive, and quality of life related outcomes in older adults with dementia: a study protocol of a randomized controlled trial

**DOI:** 10.1186/s12877-015-0024-1

**Published:** 2015-03-18

**Authors:** Johanna G Douma, Karin M Volkers, Jelle Pieter Vuijk, Marieke H Sonneveld, Richard HM Goossens, Erik JA Scherder

**Affiliations:** 1 VU University, Department of Clinical Neuropsychology, Van der Boechorststraat 1, 1081 BT Amsterdam, the Netherlands; 2 TU Delft, Faculty of Industrial Design Engineering, Landbergstraat 15, 2628 CE Delft, the Netherlands; 3 Erasmus MC, Department of Neuroscience, Dr. Molewaterplein 50, 3015 GE Rotterdam, the Netherlands; 4 University of Groningen, Center for Human Movement Sciences, Antonius Deusinglaan 1, 9713 AV Groningen, the Netherlands

**Keywords:** Older adults, Dementia, Mirror neuron system, Action observation, Physical activity, Cognition, Quality of life, Intervention, Randomized controlled trial

## Abstract

**Background:**

The number of older adults with dementia is expected to increase. Dementia is not only characterized by a decline in cognition, also other functions, for example, physical functioning change. A possible means to decrease the decline in these functions, or even improve them, could be increasing the amount of physical activity. A feasible way hereto may be activation of the mirror neuron system through action observation. This method has already been shown beneficial for the performance of actions in, for example, stroke patients. The primary aim of this study is to examine the effect of observing videos of walking people on physical activity and physical performance, in older adults with dementia. Secondary, effects on cognition and quality of life related factors will be examined.

**Methods/Design:**

A cluster randomized controlled trial is being performed, in which videos are shown to older adults with dementia (also additional eligibility criteria apply) in shared living rooms of residential care facilities. Due to the study design, living rooms instead of individual participants are randomly assigned to the experimental (videos of walking people) or control (videos of nature) condition, by means of drawing pieces of paper. The intervention has a duration of three months, and takes place on weekdays, during the day. There are four measurement occasions, in which physical activity, physical functioning, activities of daily living, cognition, the rest-activity rhythm, quality of life, and depression are assessed. Tests for participants are administered by a test administrator who is blind to the group the participant is in.

**Discussion:**

This study examines the effect of the observation of walking people on multiple daily life functions and quality of life related factors in older adults with dementia. A strength of this study is that the intervention does not require much time and attention from caregivers or researchers. A challenge of the study is therefore to get to know for how long residents watch the videos. However, the design implies a high feasibility of the study, as well as a high applicability of the intervention into daily care.

**Trial registration:**

NTR4708. Date of registration: 31 July 2014.

## Background

During the second half of the last century, there was a vast increase in the number of people aged 60 and above, from 205 million people worldwide in 1950 to 606 million people in 2000 [1]. This number is expected to increase to almost two billion in 2050. Within this group of older adults, the group of people aged 80 and above is increasing the fastest [2]. As age is an important risk factor for dementia [3], an increase is also expected in the number of people with dementia, from about 35.6 million people in 2010, to 115.4 million in 2050 [4]. One of the main characteristics of dementia is a progressive cognitive decline [5]. In the most prevalent subtypes of dementia, Alzheimer’s disease and vascular dementia [6], cognitive functions such as memory and executive functions (EF) already decline in an early stage of the disease, although in a different degree for both subtypes [7].

With the decline in cognitive functions, the dependency of people with dementia increases [5]. Indeed, for many subtypes of dementia, a decline in cognitive functions is associated with increasing limitations in activities of daily living (ADL) [8]. Other aspects of daily life functioning, such as physical functioning, are also altered in dementia. Functional mobility and muscle strength already decline over the adult lifespan [9], and physical functions such as mobility and lower extremity strength decline even more in people with dementia [10]. In both older adults and people with dementia also several gait disturbances are present [11]. Depending on the presence and subtype of dementia, these gait disturbances are, for example, wide base, decreased velocity, and decreased step length. Additionally, the rest-activity rhythm becomes weaker and more fragmented over the adult lifespan [12]. In people with dementia, many sleep disturbances such as a disrupted sleep-wake rhythm are present [13].

Because of the negative influences of dementia on multiple aspects of daily life, and no cure for dementia is available yet [14], it is of clinical relevance to find a way to decrease the decline in these functions or even improve them. This could be achieved by an increase in physical activity. Physical activity has been shown to have multiple beneficial effects for older adults in general. Not only is physical activity an important factor in maintaining health [15], it has also been found to be beneficial for mobility/physical functioning [16], to lower the risk of (progression of) ADL disability [17], to reduce depressive symptoms in older adults with depression [18,19], and, although based on only one study of 10 male participants, to influence the rest-activity rhythm by reducing its fragmentation [20]. Exercise may also have beneficial effects on several aspects of cognition, but the findings for this outcome measure are more equivocal [21,22]. The same holds for quality of life (QoL), on which beneficial effects have also been found in some but not all studies [23].

Despite the numerous beneficial effects of physical activity, many older adults are insufficiently active. Even though the percentage of older adults that meets the recommended physical activity level varies widely across studies, most of the reported percentages do not exceed 50% [24]. The level of inactivity is higher in older adults than in younger age groups [25], and people living in nursing homes or other residential care facilities seem to have even higher levels of inactivity than their community-dwelling counterparts [26-28]. Many people with dementia relocate to a nursing home, which implies they are at a higher risk of becoming sedentary. Being physically active is however not only found to be effective for older adults in general, also older adults with cognitive decline or dementia may experience beneficial effects from physical exercise. Beneficial effects have been reported on for example, mobility/physical functioning [29], ADL [10] and cognitive function [22]. However, for cognitive function findings were more equivocal [22].

Because of the beneficial effects of physical activity, it is important to motivate older people with dementia to become more physically active. Of all physical activities, walking is among the easiest to perform in daily life [30]. Many people are able to perform this activity, which makes walking a suitable activity to encourage older adults to do. However, it is time-consuming for caregivers to go for a walk with residents on a regular basis. Moreover, considering the expected increase in the number of older adults [1], as well as in the ratio of older adults to the working-age population [31], it will most likely become harder for caregivers to find time to motivate older people for and supervise them in the participation in a walking activity. A more feasible and less demanding way to increase walking and obtain its beneficial effects in older adults with dementia may be activation of the mirror neuron system (MNS) through action observation.

Mirror neurons become activated both when people perform an action themselves, and when they see someone else perform the same action [32]. The MNS seems to become activated only if an observed action exists in one’s own motor repertoire [33], and it becomes activated more if the specific motor skill observed is acquired better, that is, expertized [34]. Clearly, for ambulatory people, walking is an action that meets both these ‘requirements’. Walking can be considered a voluntary movement, as opposed to an affective movement. The MNS that is activated in the recognition of voluntary movements, the parietofrontal MNS, consists of the parietal lobe, the premotor cortex (PMC), and the posterior inferior frontal gyrus [33]. Both the PMC and the parietal lobe are activated in a somatotopic way [35]. Areas that are activated during observation of foot movements related to an object (e.g., ball kicking), are the dorsal sector of Brodmann’s area 6, and posterior parts of the parietal lobe (Brodmann’s areas 7 and 39). In addition, during observation of a person walking corticospinal excitability has been found to increase [36].

Observation of an action seems to have a facilitative effect on the execution of the observed action, as has been found for the movement onset of finger movements [37]. Furthermore, activation of the MNS through action observation has been shown beneficial, for example, for the rehabilitation of motor functions after stroke [38]. In the described studies, participants that both observed and executed a specific action (i.e., action observation therapy), showed a larger improvement in the function concerned than participants who performed the action without observing it. Action observation therapy has also been shown to decrease the number of freezing of gait episodes in patients with Parkinson’s disease for a longer period of time than practicing the action and watching images of landscapes [39]. Another pilot study showed a greater improvement in daily actions in patients with Parkinson’s disease who received action observation therapy, than in patients who performed the actions and watched videos in which no physical movements were shown [40].

Based on among others the activation of the PMC and the corticospinal excitability induced by observing an action, observing a certain movement (e.g., walking) may facilitate people to initiate the execution of that movement. This may increase their amount of physical activity. Furthermore, based on the effects of action observation therapy on the execution of the observed action, and the beneficial effect of physical activity on physical functioning and ADL, these physical performance measures may also improve. This would imply that also the quality of movements may improve as a result of action observation. Thus, the primary hypothesis in this study is that the observation of videos of walking people will have a beneficial effect on the amount of physical activity (walking) as well as on physical performance (the ‘quality’ of movements, e.g., physical functioning and ADL) in older adults with dementia. In addition, secondary effects may follow directly from action observation and/or indirectly through an increase in physical activity. The secondary hypothesis, therefore, is that the observation of videos of walking people has beneficial effects on cognition and QoL related outcomes (i.e., the rest-activity rhythm, QoL and depression). In sum, this study aims to examine the effects of observation of walking on physical, cognitive, and QoL related outcome measures in older adults with dementia.

## Methods/Design

### Study design

Reporting the design of this study follows the CONSORT guidelines [41] as strict as possible, except for the guidelines for the results and discussion sections, as this is a study protocol. This study is a cluster randomized controlled trial (RCT). The data is hierarchically ordered with participants (level 2) nested within living rooms (level 3) and four measurement occasions per participant (level 1). Since randomization at level 2 is not possible due to the study design, block randomization is being performed at level 3 where for each set of two living rooms of residential care facilities, the living rooms are randomly assigned to the experimental or control condition, thereby taking into account an evenly divided number of living rooms per condition. When a single additional or separate living room is included, and an even number of living rooms has been included before, this single living room is randomly assigned to the experimental or control condition. However, when an odd number of living rooms has been included before, the single living room is assigned to the condition that occurred least before. Randomization at level 3 is necessary since residents of the control condition should not be able to see the videos of the experimental condition, and vice versa. The complete randomization process is performed by the researcher, and randomization occurs by drawing pieces of paper. These pieces of paper are equally sized and folded, so that the drawing process occurs blindly.

### Setting

This study takes place in residential care settings, such as nursing homes. Due to the design of the study, these care settings need to have at least one living room in which residents with dementia are present during the day. The care settings are located in the Netherlands, and optionally in other European countries.

### Participants

The participants should be older adults (aged 70 years and older) with a diagnosis of dementia (as stated in their medical files). In addition, they should have a score of ≤25 on the Dutch version of the Mini-Mental State Examination (MMSE) [42]. This score is indicative of at least mild cognitive decline. This criterion was changed after trial commencement, from the preceding inclusion criterion of an MMSE score of 15–25. It was decided that residents with a lower score could also be included, as most outcome measures can be assessed for them as well. Only for cognitive functioning a shortened neuropsychological test battery is used for participants with an MMSE score <15 (see ‘Measurement occasions’ under the heading ‘Procedure’). For inclusion it is furthermore important that participants are usually present in the living room during the day.

Exclusion criteria are: a history of alcoholism, cerebral trauma, hydrocephalus, neoplasm, history of depression, personality disorders (other than those based on dementia), disturbances of consciousness, not being ambulant, and visual impairments. The last-mentioned exclusion criterion should be interpreted in such a way that participants should be able to see the videos, and to see visual stimuli during the test assessments. For the test assessments, it is also important that participants’ hearing is sufficient.

The following possible confounders are registered if available: subtype of dementia, age, gender, education level (using the Verhage system [43], with scores ranging from 1 = less than six grades of primary education to 7 = university), the use of visual, hearing, or walking aids (including the type), comorbidity, and medication use.

### Power calculation

A power analysis for a 2 x 4 mixed factorial design with two groups and four measurement occasions, with α = .05, β = .80, and an effect size of f(V) = .25, resulted in *n* = 179 participants. Taking into account a dropout of 10%, this results in a total of *n* = 199 participants. This power calculation was performed using G*Power 3.1.4 [44].

### Procedure

**Figure 1:**
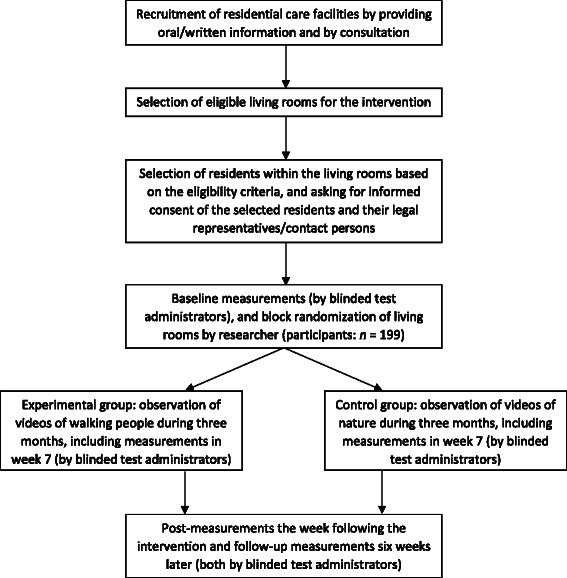
**Overview of the study protocol.**

For an overview of the study protocol, see Figure 1.

#### Inclusion of participants

The selection of residents is based on the criteria mentioned under the heading ‘Participants’, which are verified by means of the medical files of the residents, in consultation with the medical or nursing staff. The selected residents are asked whether they would like to participate, and their legal representative/contact person is contacted to ask for his/her permission as well. Subsequently, the MMSE is administered; if the score is ≤25, and if the person’s vision and hearing is sufficient based on observations during this test, written consent is asked from the legal representative/contact person and from the resident. When at least two residents in a living room agree to participate, the living room can be included in the study by the researcher.

#### Intervention

The intervention consists of videos being shown in the living room of a residential care facility. In the experimental condition, videos of people walking through different environments are shown; in the control condition, videos of nature and buildings are shown. All videos are made especially for research purposes, and are without sound. There are 10 videos of half an hour each available per condition. For each condition, a compilation of the concerning videos is made, so that the videos can easily be shown successively. The videos are shown on 42 inch Smart TVs. The loop function on these televisions is selected, in order to show the compilation videos continuously. The videos are shown on two television screens per living room, placed in such a way that the videos are visible for all participants from their usual seats. The intervention is an addition to the regular living room setting, that is, if there is already a television present in a living room, that television may stay and the two televisions for the intervention are added.

The intervention has a duration of three months (a recommended minimum duration for physical activity interventions [10]), and takes place five days per week, from Monday to Friday. At the intervention days, the videos should be switched on at the beginning of the day, and switched off at the end of the day. They may also be switched off earlier, if residents move to other rooms earlier during the day.

#### Treatment exposure

In order to know how long the participants were able to watch the videos, the care workers are asked to write down the time the televisions are switched on and off, and, additionally, to write it down if a participant was not present in the living room, for example, due to illness. Caregivers can also write down anything noteworthy about a particular participant, or something more general being observed.

In order to make an estimation of the treatment exposure, observations are being made by the researchers to register how often and how long the participants watch the videos. Per living room, there are three observation shifts during the entire intervention: one from the moment the participants sit in the living room to lunch, one from the start of lunch to diner, and one from the start of diner to the moment the participants have left the living room. The three observation shifts are planned on three separate days.

To determine the total time participants watch the videos, participants’ watching behavior is observed by means of behavior sampling [45]. Each time a participant looks at the screen, the duration of this behavior is written down. However, since multiple participants have to be observed at the same time by one researcher, the observed behavior may be missed at times. For a more detailed observation per participant, each participant is observed individually for one minute per hour, by means of focal sampling [45]. At the start of each hour within an observation shift, all participants are observed successively. Different from the recommended procedure [45], the order in which the participants are observed is kept constant. Similar to the observation order applied in other studies [46,47], the researcher observes the participants starting from the participant sitting most left, continuing towards the participant sitting most right from the researcher.

After a full observation shift, multiple scores of the total time each participant has watched the videos are calculated. The total time is calculated from the behavior sampling procedure and the focal sampling procedure separately, and from the combination of both procedures. The same holds for the number of times each participant has watched the videos. Additionally, the total time the participant is present in the living room during the shift is registered.

#### Measurement occasions

**Figure 2:**
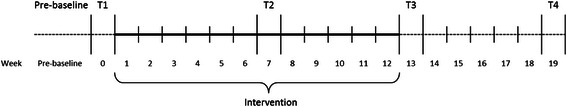
**Overview of the intervention and measurement occasions.**

The week before the intervention, baseline measurements take place (T1). The same measurements take place halfway through the intervention, being the seventh week of the intervention (T2), the week immediately after the intervention (T3), and six weeks after T3, during a follow-up week (T4). See Figure 2 for an overview of the complete study period.

During all four measurement occasions, measurements and several test administrations take place (see Table 1). The physical tests are administered by two test administrators and the neuropsychological tests and the QoL questionnaire by one test administrator. All these test administrators are blind to the study condition of the participant.

**Table 1:** **Measurements and Test Administrations at T1, T2, T3, and T4 for Participants and Caregivers**

	Participant		
Measures	MMSE 15-25	MMSE <15	Caregiver
**Primary**			
***Physical activity***			
Actometer, worn 24/7 (M10)	X	X	
***Physical performance***			
*Physical functioning*			
TUG (+sensors)	X	X	
4MWS (+sensors)	X	X	
STS	X	X	
*ADL*			
Katz ADL			X
**Secondary**			
***Cognition***			
MMSE	X^a^	X^a^	
8WT	X		
Digit Span	X	X	
VMS	X		
Face recognition	X		
Picture recognition	X		
Picture completion	X	X	
Letter fluency	X		
Category fluency I (animals)	X	X	
Category fluency II (professions)	X	X	
***QoL related***			
*Rest-activity rhythm*			
Actometer, worn 24/7 (IS, IV, RA, M10, L5)	X	X	
*QoL*			
DQoL	X	X	
QUALIDEM			X
*Depression*			
Cornell Scale			X

*Note*. MMSE = Mini-Mental State Examination; M10 = Most active period of 10 hours; TUG = Timed Up and Go test; 4MWS = Four Meter Walking Speed test; STS = Sit to Stand test; ADL = Activities of daily living; 8WT = Eight words test; VMS = Visual Memory Span; QoL = Quality of life; IS = Interdaily Stability; IV = Intradaily Variability; RA = Relative Amplitude; L5 = Least active period of five hours; DQoL = Dementia Quality of Life.

^a^Administration pre-baseline used as score at T1.

The complete neuropsychological test battery including the QoL questionnaire has a duration of approximately 90 minutes. For participants with an MMSE score <15, a shortened neuropsychological test battery is administered in order to still be able to detect possible changes in cognitive functioning, without the need for too much effort of the participant (see Table 1). All other outcome measures are the same for all participants. The shortened neuropsychological test battery including the QoL questionnaire has a duration of approximately 40 minutes. All measurements and tests are described in more detail under the heading ‘Materials’.

### Materials

#### Primary outcome measures

##### Physical activity

Physical activity is measured by means of an actometer, the Actiwatch (AW)2 (Respironics Inc., Murrysville, PA, USA). The actometer has the shape of a watch, and is worn on the wrist of the dominant arm. It is worn 24 hours per day, for a period of seven days. Caregivers are asked to temporarily take off the actometer when a participant takes a shower, goes swimming, or performs another activity in which the actometer could be exposed to too much water.

The actometer measures the arm movements of the participant; based on these movements, the rest-activity rhythm and physical activity are determined. The epoch length used is 1.00 minute. Data are analyzed by means of the PC-program Respironics Actiware 6 (Respironics Inc., Murrysville, PA, USA). Hereafter, five parameters are calculated: Interdaily Stability (IS), Intradaily Variability (IV), activity during the Most active period of 10 hours (M10), activity during the Least active period of five hours (L5), and Relative Amplitude (RA). Additional, more detailed, information on these parameters can be found under the heading ‘Rest-activity rhythm’. The parameter M10 is used to determine the amount of physical activity.

##### Physical performance

Physical functioning To assess physical functioning, three tests are administered. First, the Timed Up and Go test (TUG) [48] is administered. The chair being used for this test should have a seat with a height of 50 centimeters, the seat should be rather hard, and the chair should have armrests. The participant starts sitting in the chair, with his/her back against the backrest. The participant is then asked to stand up from the chair, walk a previously marked distance of three meters, turn around, walk back, and sit down again. The time is measured from the moment the participant starts the test, until his/her back is against the backrest again.

Secondly, the Four Meter Walking Speed test (4MWS) [49,50] is administered, using a slightly adapted protocol (i.e., each of the two conditions of the test is administered once instead of twice). In the first condition of the test, the participant is asked to stand at one spot, and walk a previously marked distance of four meters at his/her preferred speed. In the second condition, the participant is asked to do the same, except now the instruction is to walk the distance as fast as possible. For both conditions, the time it takes the participant to walk the four meters is recorded, as well as the number of steps taken. The mean walking speed and mean number of steps of these two conditions are used as outcome measures.

For these two physical functioning tests, the participants are allowed to use their regular walking aid. Additionally, during the performance of both tests, participants wear two sensors, one attached to the instep of each foot with an elastic strip. The sensors, EXLs1 (EXEL s.r.l., Bologna, Italy), are inertial sensors, equipped with a gyroscope, a magnetometer, and an accelerometer. The data from the sensors is sent to the tablet-PC program μSensorData (EXEL s.r.l., Bologna, Italy) through Bluetooth. For the purpose of the gait patterns analysis, the start and end of each test or condition of the test are marked in the data stream. Gait patterns that can be extracted are velocity and step length. At this moment, it is examined whether it is possible to extract additional gait patterns, such as shuffling gait and wide base.

Lastly, the Sit to Stand test (STS) [51] is administered. The chair being used in this test has to meet the same requirements as the chair being used for the TUG. For the STS, the participant sits down on a chair, and is then asked to stand up and sit down as many times as possible within 30 seconds. Contrary to the original STS, the participant may use his/her arms to stand up and sit down (see also [52]). The total number of times the participant sits down is the score on the test. If a participant ends standing, this counts as an additional half point.

##### Activities of daily living

To assess ADL, the Dutch translation of the Katz ADL [53] is used. This is a questionnaire on how (in)dependent a participant is in the execution of activities of daily life, such as getting dressed. The scale exists of six items which can be scored from 1–3: 1 being completely independent in the activity concerned, and 3 being very dependent. The maximum score on the test is 18, which is indicative of the participant being most dependent. The questionnaire is filled out by a caregiver.

#### Secondary outcome measures

##### Cognition

Global cognition To assess cognition, a neuropsychological test battery is administered. One of the administered neuropsychological tests is the Dutch translation of the MMSE [42]. The MMSE is a test that measures several aspects of cognition, and is therefore used as a test for general cognition. The maximum score on this test is 30, which indicates good cognitive functioning.

##### Short-term memory

The Eight words test (8WT), a subtest from the Amsterdam Dementia Screening test [54], is administered to measure several aspects of memory. First, a list of eight words is read to the participant, and he/she is asked to recall as many of these eight words as possible. The list is then read four more times, and after each time, the participant is again asked to recall as many words as possible, including the words he/she already mentioned in an earlier trial. This subtest, immediate recall, is administered to measure auditory/verbal short-term memory. The score on this test is the total number of correctly recalled words (maximum score: 40).

For the Digit Span test, a subtest of the Wechsler Adult Intelligence Scale III [55], a sequence of digits is read to the participant. In the forward condition of this test, the participant is asked to repeat these digits in the same order. The test starts with two digits in a row, and after three items of the same length, the number of digits increases with one. The largest sequence consists of eight digits in a row. The test is ended earlier if a participant incorrectly repeats at least two items of the same length, but all three items of this length need to be administered. This condition measures auditory/verbal short-term memory. The number of correctly repeated items is the score on this subtest (maximum score: 21).

Finally, also the Dutch version of the Visual Memory Span (VMS), a subtest from the Wechsler Memory Scale [56] is administered. In the forward condition, which measures visual/spatial short-term memory, the participant is shown a card with a pattern of eight squares. He/she is then instructed to tap these squares in the same order as the test administrator demonstrated. The test starts with a sequence of two squares, and after each pair of items of the same length, one square is added. The most difficult sequences contain eight squares in a row. The test is ended earlier if the participant taps both items of the same length incorrectly. The total number of items tapped correctly is the score on this condition (maximum score: 14).

##### Long-term memory

After the immediate recall of the 8WT, there is a delay of about 10–15 minutes in which other, non-memory tests are administered. After this delay, a delayed recall subtest of the 8WT is administered, in which the participant is asked to recall as many words as possible of the eight words mentioned earlier. This subtest measures long-term auditory/verbal long-term memory. The total number of correctly recalled words is the score on this subtest (maximum score: 8).

Following the delayed recall, the recognition subtest of the 8WT is administered, in which the participant is read a list of 16 words. Eight of these words were mentioned earlier, whereas the other eight are new words. The participant has to decide for each word whether it was part of the list mentioned earlier or not. This subtest also measures auditory/verbal long-term memory. The score is the number of correct answers (maximum score: 16).

Two tests of the Rivermead Behavioural Memory Test [57] are administered to measure visual long-term memory. These are Face recognition and Picture recognition. During Face recognition, the participant is shown five cards on which faces are displayed. The participant is instructed to pay close attention, in order to recognize these faces better afterwards. To help the participants pay attention, they are instructed to indicate at each face whether the person displayed is a man or a woman. Each face is shown for five seconds. After a delay of a few minutes, in which a non-memory test is administered, the participant is shown 10 cards with faces. Five of these cards were shown before, whereas the other five were not. The participant is then instructed to say whether the face being displayed is one of the previously shown faces. The score on this test is the number of correct answers minus the number of incorrect answers. The possible scores range from −10 to 10.

The test Picture recognition follows the same protocol as Face recognition, except now first 10 pictures are shown of which the participant has to indicate what the item displayed is. After the delay of a few minutes, in which again a non-memory test is administered, 20 pictures are shown. Of these 20 pictures, 10 were shown before, whereas the other 10 were not. The score is the number of correct answers minus the number of incorrect answers, and ranges from −20 to 20.

##### Visual integration

Picture completion, a subtest from the Groninger Intelligence Test (GIT) [58], is administered to measure visual integration. The participant is shown incomplete pictures, and is asked what is being shown on the picture. If the participant gives an incomplete description, the test administrator asks him/her to describe everything he/she sees. There are 22 pictures in total, of which the first two are examples. The test is ended if the participant has given five wrong answers in a row. The score is the total number of correctly recognized pictures (maximum score: 20).

##### Executive functions: fluency

The Letterfluency test [59] is administered to measure word fluency. In this test, the participant is instructed to name as many words as possible in one minute, all starting with the same letter. He/she is not allowed to say names of people or places, numbers, or successive words starting with the same prefix. First, the participant is asked to name some words with an example starting letter. Then the real test is administered, using the letters ‘D’, ‘A’, and ‘T’. If a participant remains silent for 15–20 seconds, the test administrator helps him/her by giving examples. If necessary due to incorrect answers, instructions may be repeated in between two letters. The score on this test is the sum of the number of correctly named words for each letter.

To measure category fluency, two subtests from the GIT [58] are administered: Category fluency I and Category fluency II. In Category fluency I, the participant is instructed to name as many animals as possible in a period of one minute. The score is the total number of animals mentioned, in which an animal that is mentioned more than once, is counted only once. In Category fluency II, the participant is instructed to name as many professions as possible in one minute. Again, the score is the number of professions mentioned, and the professions mentioned more than once, are counted only once.

##### Executive functions: working memory

The backward condition of the Digit Span test measures auditory/verbal working memory. The protocol for this condition is the same as for the forward condition, except the digits now have to be repeated in reverse order. Again, the number of correctly repeated items is the score on this subtest (maximum score: 21).

The backward condition of the VMS measures visual/spatial working memory. For this condition, the protocol is the same as for the forward condition. The only difference is that the squares now have to be tapped in the reverse order from the order in which the test administrator tapped them. Again, the score is the total number of items tapped correctly (maximum score: 14).

##### Quality of life related factors

Rest-activity rhythm The rest-activity rhythm is measured by means of the actometer, as described earlier in this section under the heading ‘Physical activity’. The above-mentioned five parameters IV, IS, M10, L5, and RA are used to determine the rest-activity rhythm, and will here be described in more detail.

IV is used to determine the rhythm’s fragmentation, thus transitions between activity and rest within 24 hours [60,61]. Scores for IV range from 0 (a little fragmented rhythm) to 2 or higher (a highly fragmented rhythm). IS is used to determine the stability between days [60]: the coupling of the rest-activity rhythm to ‘Zeitgebers’, described by Moore-Ede et al. (as cited in [61]). Scores for IS range from 0 (low stability between days) to 1 (high stability between days), derived from Moore-Ede et al. (as cited in [61]) and elsewhere [60]. M10 is the mean activity during the 10 most active hours during the day, and L5 the mean activity during the five least active hours [61]. RA, the amplitude of the rhythm, is calculated from M10 and L5 [20]. Formulas for IV, IS, and RA can be found elsewhere [60].

##### Quality of life

To assess QoL, two questionnaires are used. One of these questionnaires, the Dutch translation [62] of the Dementia Quality of Life (DQoL) instrument [63], is incorporated in the neuropsychological test battery. The test consists of 29 questions on five different categories of QoL, and one additional question on the participant’s overall QoL. The categories that are measured using the DQoL are self-esteem, positive affect/humor, negative affect, feelings of belonging, and sense of aesthetics. After answering three trial questions, of which the participant needs to answer at least two correct to continue the test, the 30 questions are asked. For all questions, the participant is asked to provide an answer on a 5-point Likert scale. There are three different Likert scales, each used for another selection of questions. As there is a different number of items per above-mentioned category, also the maximum score per category differs [64]. For all categories except negative affect, a higher score means a higher QoL.

The second questionnaire on QoL, the QUALIDEM [65], is filled out by a caregiver. The QUALIDEM consists of 37 questions on nine different categories, and three additional questions for further research [66]. The categories are care relationship, positive affect, negative affect, restless tense behavior, positive self-image, social relations, social isolation, feeling at home, and having something to do. Caregivers can answer the questions on a 4-point Likert scale. As with the DQoL, the number of items per category differs, and therefore also the maximum score per category differs. For each category, a higher score means a higher QoL.

##### Depressive symptoms

To assess the number of depressive symptoms, the Cornell Scale for depression in dementia [67] is filled out by a caregiver [68]. This questionnaire consists of 19 items on five categories: mood related signs, behavioral disturbance, physical signs, cyclic functions, and ideational disturbance. The questions have to be answered on a 4-point Likert scale, of which one option is ‘unable to evaluate’. For this test a total score over all items is calculated. The higher this score, the more depressive symptoms (maximum score: 38).

##### Statistical analysis

First, to compare the two groups regarding participant characteristics, independent sample *t*-tests or Mann–Whitney *U* tests will be used, depending on the distribution, for age, education level, the number of comorbidities, and the number of medicines used. Chi-squared tests will be performed to compare the groups on gender, subtype of dementia, the use of visual, hearing, or walking aids, the presence of (types of) comorbidities, and the use of (types of) medication. If the two groups differ significantly on a participant characteristic, then this variable can be included as a covariate in the hierarchical mixed model analyses.

Group comparisons will also be made for all the baseline values of the physical, cognitive, and QoL related outcome measures, to ensure equality of the groups at the beginning of the intervention. This will be done by means of an independent sample *t*-test or Mann–Whitney *U* test, depending on the distribution.

Hierarchical mixed model analyses will be used to examine the effect of the intervention on primary (physical), and secondary (cognitive, and QoL related) outcomes. For all physical outcome measures (physical activity, the physical functioning tests, the gait patterns, and ADL) mixed models will be fitted with living room as a level 3 variable, participant as a level 2 variable, and, nested within participants, time of measurement as a level 1 variable. Explanatory variables are time of measurement (four levels with baseline as the reference category) and group (experimental and control condition). A significant interaction effect is indicative of a treatment effect.

All the cognitive outcome measures will be transformed into *z*-scores. Then, a factor analysis will be performed in order to examine whether domains can be formed (e.g., a memory domain). A Cronbach’s alpha of α = .70 will be considered sufficient [69]. Subsequently, both for the possible domains, as for the separate neuropsychological tests (using the raw scores for the latter), mixed models will be fitted. The same levels and explanatory variables apply as for the physical outcome measures. Again, a significant interaction effect is indicative of a treatment effect.

For the QoL related outcome measures (the parameters of the rest-activity rhythm, the DQoL, QUALIDEM, and Cornell Scale for depression in dementia), also mixed models will be fitted. Again, the same levels and explanatory variables apply as for the physical outcome measures, and a significant interaction effect is indicative of a treatment effect.

The analyses will be performed on an intention-to-treat basis, using the statistical software PC-program SPSS. For all described analyses, a significance level of α = .05 will be used. The appropriate Bonferroni correction will be applied to the significance level in cases of multiple comparisons within a domain.

### Ethical considerations

The Medical Ethical Committee of the VU University Medical Center approved for this study.

## Discussion

The aim of this study is to examine the effect of observing videos of walking people on physical activity, physical functioning, and ADL, as well as on cognitive functioning, the rest-activity rhythm, QoL and depression, in older adults with dementia. This is examined using an RCT design, in which videos are shown in shared living rooms of residential care facilities. Since it is not possible to have participants of both conditions in the same living room (this would require videos of both conditions being shown in one living room, causing participants of each group to see videos of both conditions), living rooms instead of individual participants are randomly assigned to the experimental or control condition.

This study has several strengths. First, the study is highly feasible. For the intervention days, the only actions that are asked of caregivers are turning the televisions on and off, and writing down a minimum amount of information. This also implicates that if this intervention has beneficial effects on one or more of the outcome measures, the intervention is easily applicable in daily life in residential care facilities, and possibly also in home care settings. The applicability is due to the little amount of time needed, that is, only for turning on and off the televisions, and also to the limited associated costs. Another strength of this study is that it gives an extensive insight in the effects of the intervention on (the quality of) daily life functions, such as physical functioning, as well as on QoL itself. Additionally, some outcome measures are measured objectively, through the use of actometers or sensors.

There are also challenges in this study. Actually, one of the advantages of the study is a challenge at the same time. Since the intervention does not require much time and attention from caregivers or researchers, they are not necessarily present in the living room all the time. It is therefore difficult to get a good idea of whether and how long residents are present in the room, and how long they watch the videos. This is overcome as good as possible, without the need for much additional time and effort, by asking caregivers to write down whether participants were present/absent in the living room during an intervention day. In addition, to know whether participants watch the videos being shown, additional observations are made by the researchers by means of behavioral and focal sampling techniques.

Another aspect to take into consideration is the large number of tests that is administrated. Especially the duration of the complete neuropsychological test battery containing the DQoL may be demanding for the participants. Therefore, test administrators are instructed to pay attention to among others fatigue of participants, and, if necessary, look for a good moment for a short break, or even postpone a part of the test administration.

Beneficial effects may obviously manifest themselves through improvements in the outcome measures. However, as partly reasoned elsewhere [52], since many of the measured functions typically decline with age and/or dementia, stabilization or a decreased decline in these functions would also be an important beneficial effect of the intervention. Indifferently how the beneficial effects will manifest themselves, this intervention is expected to be a feasible means for these beneficial effects to occur. In sum, observing videos of walking people is expected to give rise to beneficial effects on physical activity, physical performance, cognition, and QoL related outcome measures, in older adults with dementia.

## Abbreviations

4MWS: Four meter walking speed test
8WT: Eight words test
ADL: Activities of daily living
DQoL: Dementia quality of life
EF: Executive functions
GIT: Groninger intelligence test
IS: Interdaily stability
IV: Intradaily variability
L5: Activity during the least active period of five hours
M10: Activity during the most active period of 10 hours
MMSE: Mini-mental state examination
MNS: Mirror neuron system
PMC: Premotor cortex
QoL: Quality of life
RA: Relative amplitude
RCT: Randomized controlled trial
STS: Sit to stand test
TUG: Timed up and go test
VMS: Visual memory span test
